# Designing a paediatric hospital information tool with children, parents, and healthcare staff: a UX study

**DOI:** 10.1186/s12887-020-02361-w

**Published:** 2020-10-08

**Authors:** Lisa Aufegger, Khánh Hà Bùi, Colin Bicknell, Ara Darzi

**Affiliations:** (NIHR) Imperial Patient Safety Translation Research Centre (PSTRC), Imperial College London, 10 S Wharf Rd, London, W2 1PE UK

**Keywords:** Children hospital information system, User experience design, Patient and public engagement

## Abstract

**Background:**

The hospital patient pathway for having treatment procedures can be daunting for younger patients and their family members, especially when they are about to undergo a complex intervention. Opportunities to mentally prepare young patients for their hospital treatments, e.g. for surgical procedures, include tools such as therapeutic clowns, medical dolls, or books and board games. However, while promising in reducing pre-operative anxiety and negative behaviours, they may be resource intensive, costly, and not always readily available. In this study, we co-designed a digital hospital information system with children, parents and clinicians, in order to prepare children undergoing medical treatment.

**Method:**

The study took place in the UK and consisted of two parts: In part 1, we purposively sampled 37 participants (n=22 parents, and n=15 clinicians) to understand perceptions and concerns of an hospital information platform specifically design for and addressed to children. In part 2, 14 children and 11 parents attended an audio and video recorded co-design workshop alongside a graphic designer and the research team to have their ideas explored and reflected on for the design of such information technology. Consequently, we used collected data to conduct thematic analysis and narrative synthesis.

**Results:**

Findings from the survey were categorised into four themes: (1) the prospect of a hospital information system (parents’ inputs); (2) content-specific information needed for the information system (parents’ and clinicians’ inputs); (3) using the virtual information system to connect young patients and parents (parents’ inputs); and (4) how to use the virtual hospital information system from a clinician’s perspective (clinicians’ inputs). In contrast, the workshop highlighted points in times children were most distressed/relaxed, and derived the ideal hospital visit in both their and their parents’ perspectives.

**Conclusions:**

The findings support the use of virtual information systems for children, in particular to explore and learn about the hospital, its facilities, and the responsibilities of healthcare professionals. Our findings call for further investigations and experiments in developing safer and more adequate delivery of care for specific age groups of healthcare users. Practical and theoretical implications for improving the quality and safety in healthcare delivery are discussed.

## Introduction

The hospital patient pathway for having treatment procedures can be daunting for younger patients and their family members, especially when they are about to undergo a complex intervention. Children are faced with an unfamiliar environment and daily treatment routine such as long bed days, separation from friends and loved ones and having to interact with unfamiliar healthcare professionals [1]. This can negatively impact their behaviour and emotions [2], and could lead to a slower rate of physical and emotional recovery without appropriate coping strategies from their caregivers [3-5].

One way to prepare young patients for the stress levels experienced during a hospital stay are educational initiatives and interventions [6]. These interventions are aimed at improving patients’ understanding, perception and awareness around key concepts in the healthcare service and delivery process [7]. They encompass knowledge on treatment procedures, how to manage chronic diseases, and preventative initiatives explaining health risk factors and their consequences [8]. Their main goals are to enable patients to play a more active, autonomous, and informed role in their treatment process, ultimately targeting stress and anxiety reduction, improving confidence, attitude and patient satisfaction [9].

Existing healthcare designs on relieving anxiety in children have focused on therapeutic play interventions prior to and during hospitalisations and/or surgical procedures [6, 7, 10-15]. Depending on the age and cognitive development of the patient, they involve preparation plays, medical plays, distraction plays, and development plays. These focus on increasing children’s understanding of medical procedures; facilitating expression of their feelings and emotions related to hospitalization; and promoting psychosocial development and preventing regression among hospitalized children. To illustrate, medical dolls have the means to explain a medical procedure [11], while videos and virtual reality games act as a distraction prior to a procedure [7]. With the support of parents and healthcare professionals, these interventions have shown to be successful in helping young patients cope with the hospital experience [4]. However, these tools have limited benefits in preparing patients for the change in environment as they take place once the child has already been admitted to hospital.

A potential approach that provides realistic expectations and understanding of the nature of a hospital admissions and treatment processes is the usage of digital application technologies. Digital health includes integrated, sustainable and patient-centred services that allow for sharing and retrieving healthcare information, and promoting effective communication between patients and healthcare providers [16].

Recent studies in paediatric treatment and care have shown that digital technology applications, such as virtual reality exposure to elective surgery procedures as part of a preparatory routine can reduce children’s anxiety, stress, and postoperative maladaptive behaviours, including difficulties in getting to sleep, temper tantrum, or decreased appetite after surgery. These application have been demonstrated more impactful than standard care alone [17-19], and, in doing so, can make healthcare safer, more accessible, and cost-effective [20].

However, for patient-centred health information technologies to be truly meaningful, the user needs to be actively involved in the design process when creating such technology [21]. User-centred design (UCD) uses rigorous methods and iterative development processes, which allows researchers and developers to (1) explore and validate new opportunities to inform their vision on the technology; and (2) give quality to and optimize the design to support its usability [22]. UCD utilises different methods [23], such as observations, personas, interviews, surveys, stakeholder workshops, user journeys and/or similar. Supported by these methods, UCD seeks to ensure effectiveness, efficiency, and user satisfaction, and avoids cost-intensive changes and modifications following the technology development.

### Context

Building on current UCD frameworks [21], this study aims to explore the possibility of designing a virtual information system specifically designed for and addressed to young patients, their caregivers and healthcare professionals, in order to comprehensively equip them for the hospital admission and treatment process. Specifically, this study consists of two parts: In part 1, we explored the possibility of a virtual information system to enable parents to verbally and emotionally prepare their child and themselves for their child’s hospital stay. We also collected data on healthcare professionals’ perceptions of such an information system, and the extent to which it is of value to healthcare service and delivery. In part 2, we worked with children and parents to understand how the information technology can be designed, presented and delivered in an engaging, age-appropriate manner. Specifically, our overarching research questions were:

1. Part 1: What are parents’ and healthcare professionals’ perceptions of having a hospital information platform specifically designed for and addressed to children?
  - What are their concerns?
  - What do they think may be the benefits?
  - What content would they like the child to learn about?
2. Part 2: What do children wish to be informed about when being prepared for a hospital admission?
  - In previous admissions, what made them feel anxious?
  - In previous admissions, what made them feel relaxed?

Information was obtained through (1) a purposive sampled group of parents and paediatric healthcare professionals, asking them about their perceptions and concerns of having a hospital information platform specifically designed for and addressed to children; and (2) a UCD workshop, where children and parents, alongside a UX designer and the research team, reflected on children’s perceptions and opinions on a virtual hospital environment, and to create first frameworks on how such environment could look like.

#### METHODS

### Sample

In part 1, a survey was sent to a total of 37 participants (n=22 parents and 15 clinicians [nurses and consultants]) who were asked about their perceptions of having a hospital information system specifically designed for children/patients. Questions in the survey were specifically developed for this study (cf. Table 1). Based on existing recommendations [24, 25], between 10 and 15 interviews were conducted to capture meaningful information patterns within interviewees’ experiences [24]. Furthermore, we assessed the data throughout the collection process to be able to determine when data saturation was reached (i.e. data does not add new meaning). The data collection took place over a period of one month, between November and December 2019. The sample was identified via purposeful outreach activities and with support of a medical student working on the paediatric ward at Imperial College NHS Trust, London. This was achieved by, for instance, approaching consultants and parents to take part in the study, and by sending out emails and posting the link of the survey on social media platforms. Written and fully informed consent was obtained before survey completion. The selection criteria for parents consisted of having experience of the hospital admission and treatment procedure for at least one child in the past. The duration of the hospital admission was flexible and could range from a few hours to a few weeks or months. For clinicians, criteria included pervious and current work experience as clinician in the paediatric healthcare sector.

**Table 1:** Questions used to explore parents’ and healthcare professionals’ thoughts and perceptions on a virtual children information system

**Questions for parents**	**Questions for healthcare professionals**
In your opinion, what kind of information and instructions should the hospital staffs give to prepare you and your child for his/her hospital stay?	In your opinion, what kind of information and instructions would you, as a healthcare professional, need to give to a young patient to prepare for his/her hospital stay?
We are interested in developing an interactive information platform in the form of a virtual hospital for kids to explore and understand the hospital environment. The platform would give educational quizzes and tasks for children to learn about the hospital facilities, and jobs of doctors, nurses and other staffs. a. Would you have your child use this platform? b. Where do you see the benefits? Where do you have concerns?	We are interested in developing an interactive information platform in the form of a virtual hospital for kids to explore and understand the hospital environment. The platform would give educational quizzes and tasks for children to learn about the hospital facilities, and jobs of doctors, nurses and other staffs. a. What aspects of your role and the hospital environment would you like the young patients to learn about? b. Where do you see the benefits? Where do you have concerns?
To what extent do you think young patients’ online presence within this information platform should be monitored (for instance, children talking to each other as part of their activity)? *Note: we would ensure no personal data could be shared and/or exploited	As part of the information system, how do you feel about video tutorials from healthcare professionals that advice parents on how to communicate and prepare their child for the hospital stay. Which aspects from your experience do you feel parents need most advice from healthcare professionals?
Within this platform, how do you feel about connecting with other parents, in order to potentially enable to exchange thoughts, advices, and to be part of a larger support group who share the same experiences. a. Where do you see the benefits? b. Where do you have concerns? *Note: Personal experiences and stories would not be disclosed through the setup of your account or registration for this game.	Do you have any other comments?
Do you have any other comments?	

In part 2, 14 children and 11 parents took part in a 2-hour workshop with the aim to explore and create a virtual environment for children to learn about the hospital, facilities, and responsibilities of healthcare professionals. Inclusion criteria were children, between 8 and 10 years of age, who had experiences with hospital admission and treatment processes (irrespective of the health condition). This age group was chosen in line with their appropriate psychological development to provide insightful inputs on their hospital experiences whilst having pragmatic contributions to the idea of using a virtual information system to explore the hospital environment [26]. The workshop was conducted at official Imperial College London White City Invention Room, and in collaboration with the Imperial College London outreach team, who recruited participants through their various collaborations with residencies from the White City area. For the workshop, parents received £40 for their and their children’s participation.

The study was approved by the Imperial College Research Ethics Committee (19IC5541). Written and fully informed consent was obtained from all participants, including parents/guardians of the minors as well as the children themselves.

### Procedure

In part 1, we explored perceptions of having a hospital information system specifically designed for and addressed to children/ young patients, as well as where parents and healthcare professionals would like more guidance in informing and preparing children for the hospital admission and treatment. Table 1 shows the questions that we used in order to obtain information (cf. Table 1).

### Workshop

In part 2, a workshop was designed based on a UCD framework that consisted of two activities:

#### Activity 1

Divided into three parts, Activity 1 aimed to create a user journey or journey mapping [27]. Journey mapping is a way to obtain the steps that are involved in a particular procedure, which, in this case, is the experience of a hospital admission and treatment [28]. In particular, we explored the temporal aspect to the admission and episode of care (i.e. medical timeline), the events that occurred in the episode of care (i.e. medical pathway), as well as goals, constraints, and healthcare professionals that were associated with and involved in the episode of care. In Activity 1a, participants were asked to create and discuss an objective description of a hospital visit and how they felt at that particular time. We plotted a framework of events to be referenced by the participants as a starting point for discussion. In Activity 1b, participants created a timeline with the key events during a visit to a hospital, i.e. when they encountered a problem or when they were confused or stressed. We aimed to understand the aspects of the hospital visit that could be improved from the patients’ point of view. In Activity 1c, participants were asked to recall the points in time when they were most relaxed. We aimed to understand the aspects of the hospital visit that were effective and efficient for the patients.

#### Activity 2

In Activity 2, participants were given the opportunity to re-design their hospital visit and to consider what features they would like in a hospital visit. Here, we applied the approach of *Cooperative Inquiry* [29-31], to engage children in the design of new technologies, whilst also accounting for the intergenerational cooperation and partnership between adults and children [31]. Merged with participating design and human factors experts, this approach provides valuable insight that can be incorporated into a technology-based design. Using the user journey of events created in *Activity 1a-c* and a guideline, the children and their parents were asked to think of interventions and technology-based designs that would have improved their experience. For this, the participants were divided into three groups, and each group presented their ideas and initiated open discussions from the room. The workshop was observed, and audio and video recorded by two members of the research team.

## Data Treatment and Analysis

The workshop was audio recorded and transcribed by an external company. Findings from both part 1 and part 2 were documented and analysed thematically by two members of the research team. Thematic analysis is a type of qualitative research method used for identifying, analysing and reporting patterns or themes within data from an inductive, semantic, iterative approach (cf. Braun & Clarke for a detailed overview) [32-34]. In other words, it allows to work data-driven and to provide an in-depth analysis of the patterns found within and across participants. In this study, we identified patterns within the data using an inductive ‘bottom up ’approach, which is characterised by a strong link with the data, and that is absent of any aim to support a pre-existing framework or that is based on the researchers’ preconceptions. This included reading the data several times in order to get familiar with the depth and breadth of the content [35]. Next, a set of initial lists of codes from the data that appeared meaningful to the first and second author. With a clear sense of the context, the coding process enabled to organise the data into themes. Themes and codes were discussed between the first and second author throughout the data analysis. After further refinement, the data was organised with accompanying narrative and supported by quotes from participants that capture the essence of the point being demonstrated. For part 1, the analytic narrative was done in consideration of perceptions and attitudes towards a virtual information system designed for and addressed to children. Similarly, for part 2, the data was analysed thematically and synthesised narratively with the findings of the drawings from activity 1, where the data was coded according to the ways in which imagination was depicted in the drawings, and then assessed for content such as the key features of the children’s most distressing and relaxing hospital experiences.

## Results

### Part 1

**Fig. 1:**
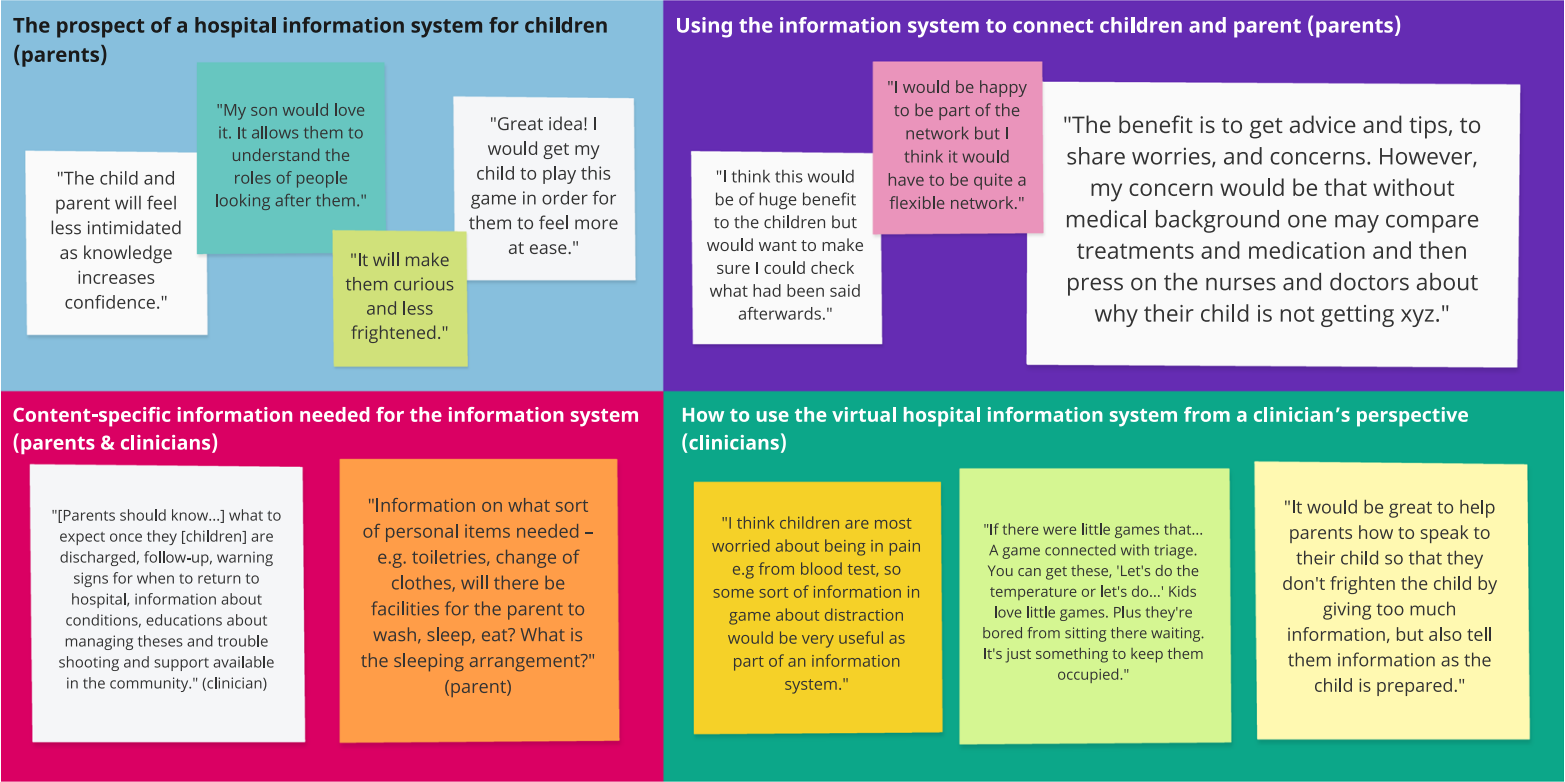
Themes and exemplar quotes from the survey of both parents and clinicians (created in Miro)

**Fig. 2:**
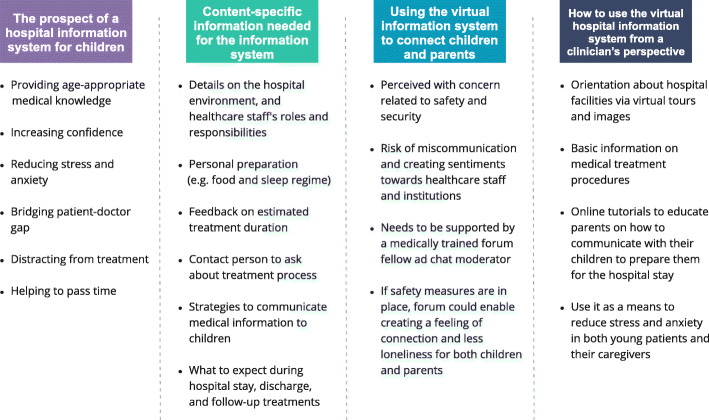
Overview and summary of the main themes (created in Miro)

Findings from parents and clinicians were categorised into four themes (cf. Figs. 1 and 2): (1) The prospect of a hospital information system; (2) content-specific information needed for the information system; (3) using the virtual information system to connect young patients and parents; and (4) how to use the virtual hospital information system from a clinician’s perspective.

- (1)*The prospect of a hospital information system for children (parents)*
  Parents were positive towards a hospital information system specifically designed for children. They acknowledged that providing age-appropriate medical knowledge and information about the hospital admission would increase their confidence, reduce stress, anxiety and fear of the unknown and bridge the patient-doctor power gap. They also emphasised that it would help children spend their waiting time more pleasantly while teaching them about the hospital in an engaging, non-threatening manner.
- (2)*Content-specific information needed for the information system (parents & clinicians)*
  Parents acknowledged the need for children to know about the hospital environment, and the roles and responsibilities of healthcare staff. Emphasis was put on information related to personal preparation at point of entry to hospital admission, i.e. whether they can stay over-night, whether they can bring personal items, what is needed, and whether and what facilities are available for them. Furthermore, they wished for an estimated timeline as to how long the admission or treatment will take, and to have information available in relation to the treatment process. Lastly, they highlighted the need for medical information that is both easily understandable for parents and conveyable to their children.
  Clinicians were keen to have an information system in place that allows patients to know about eating and sleeping arrangements, the key roles and responsibilities of staff, which staff parents and young patients can talk to if they have any questions or concerns, an introduction to the physical environment, and also an estimated timeline of how long patients will be staying to receive their particular treatment. They also felt that there was a need for advising parents on what to expect during the hospital stay, especially after discharge and for potential follow-up visits.
- (3)*Using the virtual information system to connect children and parents (parents)*
  Parents expressed concerns in relation to the possibility of online communications between young patients without any external monitoring and stressed that any online activity of their children should be subject to close parental observation. They also highlighted that security measures (e.g. a forum and chat moderator) should be in place, in order to ensure that children are interacting in a safe and secure environment.
  Similarly, the prospect of having a platform for parents to connect with each other received mixed responses. While some parents felt comforted by the idea of knowing that they are not alone, and that it would allow them to share thoughts and experiences, other parents raised concerns with regards to the lack of medical background, the risk of providing misinformation, and the possibility that the forum would cultivate unconstructive negative sentiments towards the healthcare institution and its professionals. Thus, parents advised that an online forum would need to be supported by a medically trained forum fellow, and segmented into grouped of e.g. chronic diseases, acute admission, etc., in order to allow for appropriate and context-specific support.
- (4)*How to use the virtual hospital information system from a clinician’s perspective (clinicians)*
  Healthcare professionals emphasised that the platform should be used to give orientation about the hospital facilities, displayed via virtual tours and images, and to provide some basic information on medical treatments. They also highlighted that the system should advise parents on how to effectively communicate with their child (e.g. as to what will happen during the hospital stay), in order to reduce stress levels for both young patients and their caregiver

### Part 2

**Fig. 3:**
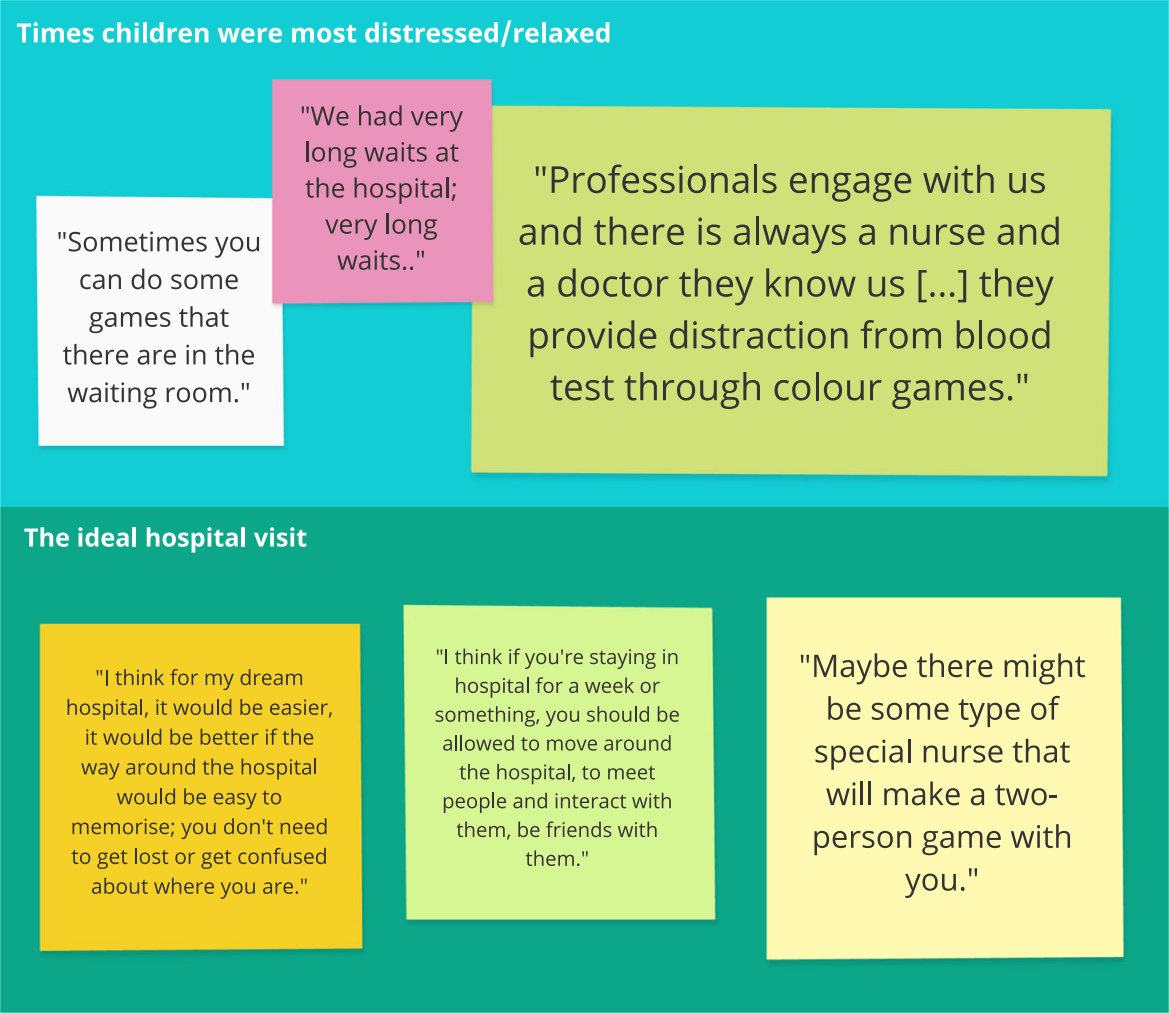
Themes and exemplar quotes from the workshop (created in Miro)

Findings from the workshop drawings and the workshop activities 1 and 2 were synthesized narratively, and themed into two categories: (1) Times children were most distressed/relaxed; and, (2) the ideal hospital visit (cf. Fig. 3).

- (1)*Times they were most distressed/relaxed*
  Distressing events were experienced throughout the treatment process (i.e. pre-arrival and arrival time, treatment). In particular, pain-points were expressed in relation to feeling lost, confused, where to find certain locations and who to ask. Participants emphasised the long waiting times and/or lack of guidance as to how long they had to wait until they were seen by a doctor. It was highlighted that hospital playrooms were only open at certain times and that they were mainly configured for very young patients.
  In contrast, children felt most relaxed when they knew that their parents could stay with them at the hospital over-night, when nurses talked with them during waiting times, when they could watch movies, get snacks or stickers (i.e. tokens of achievements) for being good patients and when they received informative and clear explanations as to what would happen next.
- (2)*The ideal hospital visit*
- The children suggested that features of their ideal (digitalised) hospital included a general understanding of the physical layout of the hospital, i.e. an awareness of what shops are in the hospitals to get snacks from, and knowledge of who to speak to if they have questions or need further information. They also wished for a mini library, a dedicated (virtual), age-appropriate, playroom, and a mood test to complete during waiting times so that the doctor is aware of how the patient feels. Lastly, children wanted their parents and relatives to stay with them for comfort, and, if they required an extended admission, to have greater levels of interaction with the healthcare staff and other patients.

## Discussion

This study aimed to explore the idea of co-producing a virtual hospital information system that (1) enables parents and healthcare professionals to verbally and emotionally prepare children for their hospital stay; and, (2) allows children be informed and eased into the hospital environment and treatment procedures in a playful, age-appropriate manner. Data was obtained and analysed from surveys on parents’ and healthcare professionals’ general perception of having a virtual information system in place specifically design for and addressed to children, and from workshop activities with children and parents.

### Summary of findings

Findings from part 1 of this study showed a strong need for information for parents and children before the hospital admission. In particular, parents wished for easily digestible, non-medical explanations as to what to expect during the treatment process, accompanied by advice for parents on how to prepare their child for their hospital stay in an age-appropriate manner. They appreciated the idea of a virtual hospital information system that explains to children more about the roles and responsibilities of healthcare professionals, and, by doing so, could give them confidence and reduce anxiety and fear of the unknown. Usage of a system for children and parents to connect with each other was treated with caution and indicated that such systems need to be closely monitored and supported by medical experts.

Results from healthcare professionals highlighted the need to prepare parents and children for any policies (e.g. food and drink regimens) and schedules that they may want to consider before going to the hospital; provide an introduction to the physical environment; explain the roles and responsibilities of healthcare professionals; and suggest an estimated timeline concerning the length of waiting times to receive their treatment. They welcomed the idea of having a virtual information system that allows parents to learn how to effectively communicate with their child, and to reduce stress and anxieties.

Lastly, outcomes from the workshop reflected findings in the survey in that children were most distressed about not knowing what to expect and waiting times before treatment procedures. In contrast, children felt most relaxed when their parents could stay with them at the hospital over-night; nurses talking to them during waiting times; watching movies; getting snacks or stickers for being good patients (i.e. token of achievements); and receiving informative and clear explanations on their treatment.

### Comparison with previous literature

Evidence suggests that patient-engaging and family-centred research leads to positive health outcomes and increased satisfaction, based on the perspectives of patients, parents, families, and health care providers [36]. A growing body of literature has also found benefits of empowering patients in decision-making, not only through the use of health information decision tools [9, 37], but by engaging them in exploratory and participatory healthcare intervention activities early on [38-40], aiding researchers in designing study protocols and choosing appropriate outcomes [41]. Unfortunately, research within the inpatient setting is rudimental and the evidence-base literature is highly limited, highlighting the need for more standardised, robust, and scientifically valid studies [42]. This study fills some of the gaps within the implementation of a patient engagement framework, specifically for young patients. Through UCD toolkits of engagement activities, we enabled a true “partnership” and a bi-directional exchange, where children, parents, and healthcare professionals took part in the design and decision-making process. Through methods such as interviews, user journeys, and cooperative inquiry, families and healthcare professionals were consulted, involved, and invited to actively collaborate [43, 44], allowing our research procedure to be participative and transparent [45].

### Further avenues for research and practice

This study provides numerous avenues for further research and practice:

Researchers are advised to investigate *how* and *which* method of information delivery for the hospital environment is best suited for this kind of tool. The development of a child consists of cognitive, social, physical, and emotional domains [46], and differs in progress depending on the their age and maturity development. Digital environments need to encourage active exploration and experimentation involving senses such as sounds, images, and words for adequate opportunities in providing guided interaction and learning about the hospital environment.

Researchers are, furthermore, encouraged to conduct design studies in consideration of cultural differences and digital literacy, such as differences in colour meaning and gestures, as well as user interfaces to assist attention and comprehension [47]. Overall, an understanding of what user interface and design features (e.g. colours, fonts, layout, navigation, level of interaction) resonates with young patients and their parents will clarify the most effective way of preparing them for a potential hospital admission and treatment.

Once fully developed and implemented, studies should be designed to understand the impact of such designed information system compared to other systems, as well as standard care alone. These may include patients’ and carers’ hospital experience satisfaction, changes in anxiety and post-operative mal-adaptive behaviour, or healthcare outcomes including recovery rate indicated by e.g. the length of hospital stay. They should also explore how the system could be used to connect patients and parents, and how to actively involve healthcare professionals during the that process.

Safety and privacy concerns for application design in digital healthcare need to be tackled [48]. Medical information technology applications that allow for the possibility of data sharing have concrete implications on the design of such application. Thus, handling of sensitive data need to go through anonymization process prior to sharing to eliminate legislative obligations and constraints.

Lastly, future work is necessary in simulating similar, repeated events at a larger scale to encourage open discourses and discussions on patient information systems designed specifically for children. These discussion could be on how virtual information systems should be used to aid with the admission and treatment process; other discussion may tackle how such information technology enhance health literacy and patient safety. Health literacy enables patients to process, understand, and make sense of health information in a way that can significantly impact their health decision and behaviours [8]. By being able to create a critical thinking approach on how patients’ own behaviours impact their health, information technologies have the potential to generate a positive and stronger long-term impact, which, for continuous treatment procedures or hospital admissions appears a valuable stream of research.

### Limitations

For the workshop activity in study 2, participants were paid to take part and the sample size was relatively small. However, while this increased the risk of a sample selection bias and limited feedback [41] it allowed for enhanced interaction and participation, which was the main aim of the workshop. Future initiatives are therefore encouraged to aim for patient involvement activities at larger scales, or through iterative design workshops, that encourage increased attendance and contributions related to improving healthcare service and delivery.

Information regarding the children’s presentation, investigation or treatment at hospital was incomplete, thus we cannot ascertain whether our sample group was representative of the wider population of child patients in this regard. Recent studies have shown that information technology/anxiety reducing interventions are designed most commonly for young patients in relation to surgery with/without general anaesthesia [17-19, 49-53], burns wound treatments [54-56], and intravenous cannulation [57]. Knowing what condition and medical service the information technology will support will be crucial to help children assist in the admission and treatment process, not only for specific conditions and healthcare settings, but also when it comes to healthcare services compared to other countries. Treatment specific and localised technologies will allow for a more accurate reflection of the patient’s experiences and care.

Findings are limited in terms of its generalisation [58]. While we believe that results can be transferred to different settings within the hospital admission and treatment processes, ranging from general health check-ups to elective surgeries, where hospital information on the infrastructure and roles and responsibilities of healthcare professionals apply irrespective of the type of health care delivered, future studies are encouraged to gather more in-depth user requirements [59]. These should be context and user specific, and further inform the design and development of such information technology.

## Conclusion

This study aims to co-produce and design a virtual information system specifically designed with and for young patients and their caregivers. Results suggest that this system needs to provide information related to roles and responsibilities of healthcare staff, and basic medical information regarding patients’ treatment, in order to increase their confidence and reduce their anxiety and fear of the hospital visit. Future studies are encouraged to further investigate how to improve patients’ experience, quality and safety of healthcare delivery, through a virtual information system for younger patients with large-scale potentials.

## Data Availability

We are unable to share data due to sensitive patient information. This is line with the ethics committee approval (Imperial College Research Ethics Committee) and it was made clear on consent forms that data would not be shared to anyone other than the researcher and immediate research team. Please contact Dr Ruth Nicholson, Imperial College London, (r. nicholson@imperial.ac.uk) to field future data request.

## Abbreviation

UCD: User-Centred Design
